# Exploration of 2D Ti_3_C_2_ MXene for all solution processed piezoelectric nanogenerator applications

**DOI:** 10.1038/s41598-021-96909-0

**Published:** 2021-08-31

**Authors:** Rahmat Zaki Auliya, Poh Choon Ooi, Rad Sadri, Noor Azrina Talik, Zhi Yong Yau, Muhammad Aniq Shazni Mohammad Haniff, Boon Tong Goh, Chang Fu Dee, Navid Aslfattahi, Sameer Al-Bati, Khatatbeh Ibtehaj, Mohammad Hafizuddin Hj Jumali, M. F. Mohd Razip Wee, Mohd Ambri Mohamed, Masuri Othman

**Affiliations:** 1 Institute of Microengineering and Nanoelectronics, Universiti Kebangsaan Malaysia, 43600 Bangi, Selangor Malaysia; 2 Faculty of New Sciences and Technologies, University of Tehran, Tehran, Iran; 3 Low Dimensional Materials Research Centre (LDMRC), Department of Physics, Faculty of Science, University of Malaya, 50603 Kuala Lumpur, Malaysia; 4 Lee Kong Chian Faculty of Engineering & Science, Universiti Tunku Abdul Rahman, Sungai Long Campus, Jalan Sungai Long, Bandar Sungai Long, Cheras, 43000 Kajang, Selangor Malaysia; 5 Department of Mechanical Engineering, Faculty of Engineering, University of Malaya, 50603 Kuala Lumpur, Malaysia; 6 School of Applied Physics, Faculty of Science and Technology, Universiti Kebangsaan Malaysia, UKM, 43600 Bangi, Selangor Malaysia

**Keywords:** Energy science and technology, Materials science, Nanoscience and technology, Physics

## Abstract

A new 2D titanium carbide (Ti_3_C_2_), a low dimensional material of the MXene family has attracted remarkable interest in several electronic applications, but its unique structure and novel properties are still less explored in piezoelectric energy harvesters. Herein, a systematic study has been conducted to examine the role of Ti_3_C_2_ multilayers when it is incorporated in the piezoelectric polymer host. The 0.03 g/L of Ti_3_C_2_ has been identified as the most appropriate concentration to ensure the optimum performance of the fabricated device with a generated output voltage of about 6.0 V. The probable reasons might be due to the uniformity of nanofiller distribution in the polyvinylidene difluoride (PVDF) and the incorporation of Ti_3_C_2_ in a polymer matrix is found to enhance the β-phase of PVDF and diminish the undesired α-phase configuration. Low tapping frequency and force were demonstrated to scavenge electrical energy from abundant mechanical energy resources particularly human motion and environmental stimuli. The fabricated device attained a power density of 14 µW.cm^−2^ at 10.8 MΩ of load resistor which is considerably high among 2D material-based piezoelectric nanogenerators. The device has also shown stable electrical performance for up to 4 weeks and is practically able to store energy in a capacitor and light up a LED. Hence, the Ti_3_C_2_-based piezoelectric nanogenerator suggests the potential to realize the energy harvesting application for low-power electronic devices.

## Introduction

Energy harvesting development has attracted increasing attention due to the search for new energy technology to fulfill the rapid increase in energy demands for low power consumption electronic gadgets. With the promising abilities to harvest random abundant mechanical energy from daily activities or environments such as walking, wind, raindrop, and vehicle pressure into electrical energy through a nanogenerator, it suggests the potential to power up wearable and portable electronic devices^1–3^. In the past few years, the nanogenerator as an alternative energy harvester has been studied in triboelectric and piezoelectric devices^4–6^. Principally, a triboelectric nanogenerator generates the electrical energy via friction that induces electrostatic charge between two electrodes^7^. Meanwhile, a piezoelectric nanogenerator produces electricity through the deformation of a piezoelectric material^4,5,8^. However, the piezoelectric nanogenerators demonstrate better performances than triboelectric nanogenerators in terms of energy conversion. Triboelectric devices show the drawbacks of inconsistent electrical output from undesirable sensitivities to static electricity, stray capacitances, and temperature changes^6^. In contrast, piezoelectricity involves permanent electric charge and results in a consistent electrical signal for a sustainable energy system^9^. The first reported piezoelectric nanogenerator in 2006 is based on ZnO nanowire in the Al_2_O_3_ substrate but the maximum output voltage was attained at 8 mV^10^ that may hinder its wide applications. The improved piezoelectric nanogenerator was achieved by incorporating lead-containing material that later had to be limited in usage due to harmful effects on the human being and environment. Therefore, in order to improve the performance of piezoelectric nanogenerator and yet an environment-friendly concern, a piezoelectric polymeric nanogenerator using polyvinylidene difluoride (PVDF) material was first conducted in 2009^11^. The advantage of this polymer material is mainly attributed to its naturally flexible properties to exploit deformations induced by small forces through pressure, bending, stretching, and mechanical vibration^12^.

In general, PVDF has three dominant phases and overall, 5 phases namely α, β, γ, δ, and ε. Among the phases, α is a thermodynamically most stable and non-polar in nature, whereas β, γ, δ-phases are mainly polar crystalline phases that exhibit piezoelectricity in PVDF. Thus far, for piezoelectric nanogenerator (PENG) purposes, β-phase is of great importance among the crystalline phases as its spontaneous polarization and piezoelectric sensitivity are higher than γ- and δ-phases^13^. The macroscopic polarization of PVDF depends on the orientation of the molecular –CH_2_/–CF_2_ dipoles in the β-phase and degree of crystallinity. PVDF in electroactive polar β-phase is highly preferable for the maximum energy harvesting due to the aligned dipoles in this phase. Because of this particularly interesting property, many efforts have been attempted to induce the electroactive β-phase in PVDF. To increase β-phase, electrical poling and stretching of the sample were conducted to transform α- to β-phase, however, this technique would change the structure of PVDF and require multiple tedious preliminary steps^14^. Hence, alternative approaches have been proposed to enhance β-phase by using high polar solvents with the addition of salts and nanofillers by introducing 2D nanomaterials fillers into the PVDF matrix^5,15^. By dissolving PVDF in the high polar solvent, the electrical output of the PENG device shows significant improvement^16^. Hexamethylphosphoramide (HMPA), dimethylsulfoxide (DMSO), and dimethylformamide (DMF) were used as the solvent to dissolve PVDF. Nevertheless, HMPA shows the greatest outcome of output performance due to its higher polarity nature^15^. The polarity originating from polar N–P and O=P bonds accelerates the rate of dissolution between PVDF molecular chains and HMPA to form an β-phase state^15^. Salt addition in a high polar solvent is found to encourage the arrangement of PVDF chains into trans-state β-phase^15^. A little amount of salt added in a high polar solvent is found to affect the consequent crystalline properties of PVDF.

2D nanomaterials that have unique physical and chemical properties have been researched as a filler in the PVDF matrix to enhance the piezoelectricity of nanogenerators. The general characteristics of 2D materials are flexibility, transparency, atomic layer thickness and high surface to volume ratio are suitable for designing of mechanically flexible, optically transparent, and miniaturized nanogenerator devices. The addition of 2D nanomaterials, or known as nanofillers into the polymer matrix is reported to improve the electrical output of nanogenerators caused by the high piezoelectric coefficient of nanofiller and improved mechanical stiffness of nanogenerator^8^. The most widely used 2D materials, graphene quantum dots, and MoS_2_ flakes have been introduced as fillers in piezoelectric matrices to improve the electrical performance of the nanogenerator^4,5,17^. Titanium carbide (Ti_3_C_2_) MXene, an emerging 2D material that was discovered in 2011 has been explored in several applications such as energy storage, non-volatile memory, triboelectric nanogenerator, and sensor^18^. Nonetheless, the incorporation of Ti_3_C_2_ in piezoelectric materials is yet to be widely explored as the nanogenerator devices than expected. Ti_3_C_2_ has drawn attention in energy harvester development attributed to its high conductivity, remarkable work function, and highly electronegative surface.

In energy storage applications, MXene improves charge storage capacitance upon ion penetration between MXene sheets because of the strong bond between metal and carbon/nitride layer^19,20^. The charge trapping capability of Ti_3_C_2_ for memory application is attributed to its 4.9 eV high work function, surface terminations, and metal vacancies of Ti_3_C_2_ nanosheets^21^. Also, the incorporation of HF-etched MXene in the triboelectric nanogenerator is found to improve the electrical performance ascribed to the highly electronegative surface of the MXene which is associated with the rich of –F group^22^. In another recent study, the MXene-based piezoelectric sensor was studied by Wang et al. and found that better dipole polarization of PVDF-TrFE is obtained because of improved conductivity resulting from the higher static electric field force^23^. The hydrophilic properties of MXene attributed to the presence of the surface terminations are reported to promote the dispersion of MXene in the water-soluble polymer^24^. In addition to the aforementioned approaches to improve the electrical performance of nanogenerators, different MXene concentrations will be explored to identify the optimum device performance. Hence, in this work, we propose to demonstrate synergistic effects by blending Ti_3_C_2_ multilayers, PVDF, and salt using high polar HMPA solvent to study the output performance of piezoelectric nanogenerators. The method of layer deposition is using all-solution processes, specifically spin-coating, and spray-coating. This method has the merit of being time-efficient, low-cost, scalable and produces compatible outcomes as compared to those expensive and complicated tools or processes.

## Experimental details

In the synthesis of Ti_3_C_2_, the following materials were used without any further purification: Ti powder (− 325 mesh, 99% purity, Alfa Aesar), aluminium powder (− 100 + 325 mesh, 99.5% purity, Alfa Aesar), titanium carbide powder (− 325 mesh, 98% purity, Sigma Aldrich), Ammonium hydrogen difluoride (reagent grade 95%, Sigma Aldrich), and sodium hydroxide (97% purity, pellets, Sigma Aldrich). The precursor MAX phase Ti_3_AlC_2_ is synthesized using a molar ratio of 1:1.2:2 of (Ti:Al:TiC) elemental powders, mixed with a pestle and mortar, followed by a thermal treatment using a tube furnace under Ar atmosphere at 1400 °C for 2 h (5 °C /min heating/cooling rate). Firstly, a solution of 2 M, NH_4_HF_2_ was prepared precisely as the main part of the wet-chemistry etching process. Afterwards, the dilution process of the ammonium hydrogen difluoride was conducted using DI water to produce 20 ml of solution, followed by magnet-stirring at 300 rpm for 1 h and room temperature using a hot plate magnetic stirrer (RCT BASIC, IKA). 1 g of Ti_3_AlC_2_ was weighed using a microbalance (Explorer series, EX224, Ohaus), followed by adding to the uniform well-prepared NH_4_HF_2_ solution. Adding the MAX phase material to the prepared solution was performed slowly as the reaction is exothermic. The MAX phase suspension in the NH_4_HF_2_ was magnet-stirred at 300 r.p.m. for 48 h and room temperature continuously to conduct the etching process. After the etching process, a dilute solution of NaOH was added slowly until the pH of the solution reached 6, and was filtered and rinsed several times with deionized water. The washing process was conducted using an ultrahigh centrifuge (Sorvall LYNX 6000, Thermo Scientific) for 4 times (each time of 10 min) at 3500 rpm. The acquired MXene were then washed with deionized water, being subsequently vacuum dried under the pressure of 100 mb at 50 °C overnight.

The exfoliated Ti_3_AlC_2_ (0.3 g) was dispersed in 100 ml DI water using a Pyrex glass bottle. The delamination process was conducted by ultrasonication (Fischer Scientific, 500 Watts, and 20 kHz) under flowing Argon for 45 min, and the probe sonication pulse time was set to 6 s ON and 3 s OFF with an amplitude of 40%. This was followed by centrifugation for 45 h at 3500 r.p.m.. After centrifugation, the supernatant comprising Mxene (Ti_3_C_2_T_X_) flakes (i.e. the stable MXene colloidal solution) was collected and vacuum dried under the pressure of 100 mb at 50 °C overnight.

To obtain Ti_3_C_2_ suspension, the Ti_3_C_2_ powder was dispersed in ethanol in different mass/volume concentration at 0.01, 0.02, 0.03, 0.04, 0.05 g/L. The suspension was then sonicated for 1 h to ensure the homogeneous dispersion of Ti_3_C_2._ For the preparation of the PVDF solution, 0.1 g PVDF powder was added into 10 mL of HMPA solvent. The PVDF and HMPA were purchased from Sigma Aldrich and Merck and used as they are. Subsequently, 5 mg MgCl_2_ purchased from Sigma Aldrich was added to the prepared PVDF solution. The solution was stirred for 1 h at 60 °C to ensure the dilution of PVDF powder and MgCl_2_. Next, PVDF solutions with salt were mixed with Ti_3_C_2_ suspension for each mass/volume concentration with the ratio of 1:1 to obtain the nanocomposite solution. The nanocomposite was marked as NC1, NC2, NC3, NC4, and NC5 for 0.01, 0.02, 0.03, 0.04, 0.05 g/L, respectively to ease the discussion.

**Figure 1:**
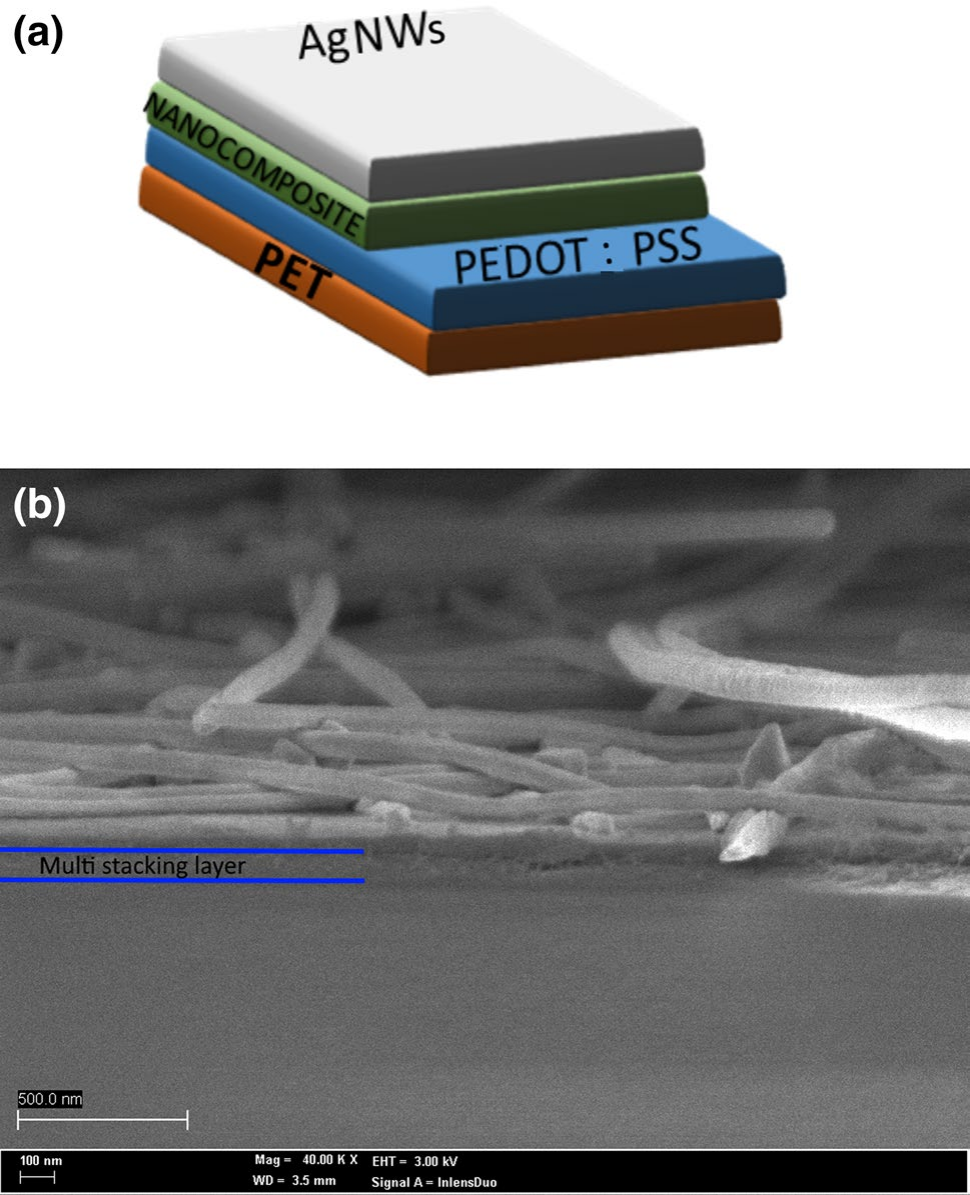
(**a**) Stacking structure design, and (**b**) corresponding FESEM cross-sectional view of the nanogenerator device.

For nanogenerator devices fabrication, silver nanowires (Ag-NWs)/nanocomposite/poly(3,4-ethylenedioxythiophene) polystyrene sulfonate (PEDOT:PSS) stacking structure were deposited on 2 cm × 2 cm polyethylene terephthalate (PET) substrate as shown in Fig. 1a. The PET was cleaned with ethanol and was treated with oxygen plasma treatment at 50 W for 5 min to improve its surface hydrophilicity. Afterward, 25 nm of PEDOT:PSS was spin-coated at 2000 rpm for 50 s on top of PET substrate to form the bottom electrode and then annealed at 80 °C for 15 min to improve its conductivity^25^. Then, the nanocomposite mixture was deposited on top of the PEDOT:PSS layer. Note that the nanocomposites with different concentrations were prepared and measured about 75 nm when spin-coated at 2000 rpm for 50 s. Then, dry it on an 80 °C hot plate for 15 min. Electrical poling of PVDF film before the electrical characterization is evitable as the spin coating deposition and drying PVDF at 80 °C were reported to encourage the formation of β-phase. Consequently, PVDF has been prepared in an enhanced polar β-phase molecular alignment state during the device fabrication process^26,27^. The device fabrication was completed by spray-coating the AgNWs in isopropyl alcohol at 0.1 MPa on top of a 70 °C hot plate to form the top electrode. The heating temperature was set below 100 °C in each deposition process to prevent heat deformation on the PET substrate due to its low thermal stability. It is worth mentioning that the 0.01, 0.02, 0.03, 0.04, and 0.05 g/L Ti_3_C_2_ concentration devices were marked as 1WD, 2WD, 3WD, 4WD, and 5WD, respectively. Figure 1b shows the field emission scanning electron microscopy (FESEM) cross-section image of the multi-stacking layer for the fabricated nanogenerator device.

X-ray diffractometer (Model: Rigaku Smartlab) with an X-ray wavelength of 1.5406 Å was adopted to study the crystalline structure. The X-ray diffractograms were recorded over a 2θ range of 20–80° at a fixed grazing incidence angle of 1.50°. The scan speed and step size were fixed at 0.50°/min and 0.01°, respectively. Infrared spectra of samples were characterized using a Perkin Elmer 2000 Fourier Transform Infrared spectroscopy (FTIR) wavenumber region between 4000 and 400 cm^−1^. The cross-sectional view of the piezoelectric nanogenerator device was observed by field-emission scanning electron microscope (Model: Cold Field UHR FESEM SU8230, Hitachi High-Technologies Corporation, Japan) at a low electron accelerating voltage of 2 kV. The morphology of MXene multilayers was investigated using a high-resolution transmission electron microscopy (HRTEM) system (JEOL, JEM-2010) at an accelerating voltage of 200 kV. The microstructure of Ti_3_C_2_ multilayers was characterized by FESEM on a Hitachi SU-8000 at an accelerating voltage of 15 kV. The working distance is set to be 15,900 μm with an emission current of 10,500 nA. The absolute transmittance of nanogenerator devices was characterized by the PerkinElmer Lambda 900 UV–VIS NIR Spectrometer. For the electrical performance of nanogenerators, open-circuit voltage (*V*_*oc*_) and short-circuit current (*I*_*sc*_) was determined using an oscilloscope, and Agilent DSO 9404A, respectively when mechanical stress was applied on the surface of the nanogenerator. The tunable mechanical stress and frequency were provided by an automatic self-assembled tapping setup to obtain the electrical behavior of the nanogenerator. The frequency and force of the tapping machine can be controlled and tuned by the Arduino circuit connected to the tapping system. The tapping force was estimated by SP-10 SHSIWI digital force gauge by varying the input voltage range from 20 to 28 V.

## Result and discussion

**Figure 2:**
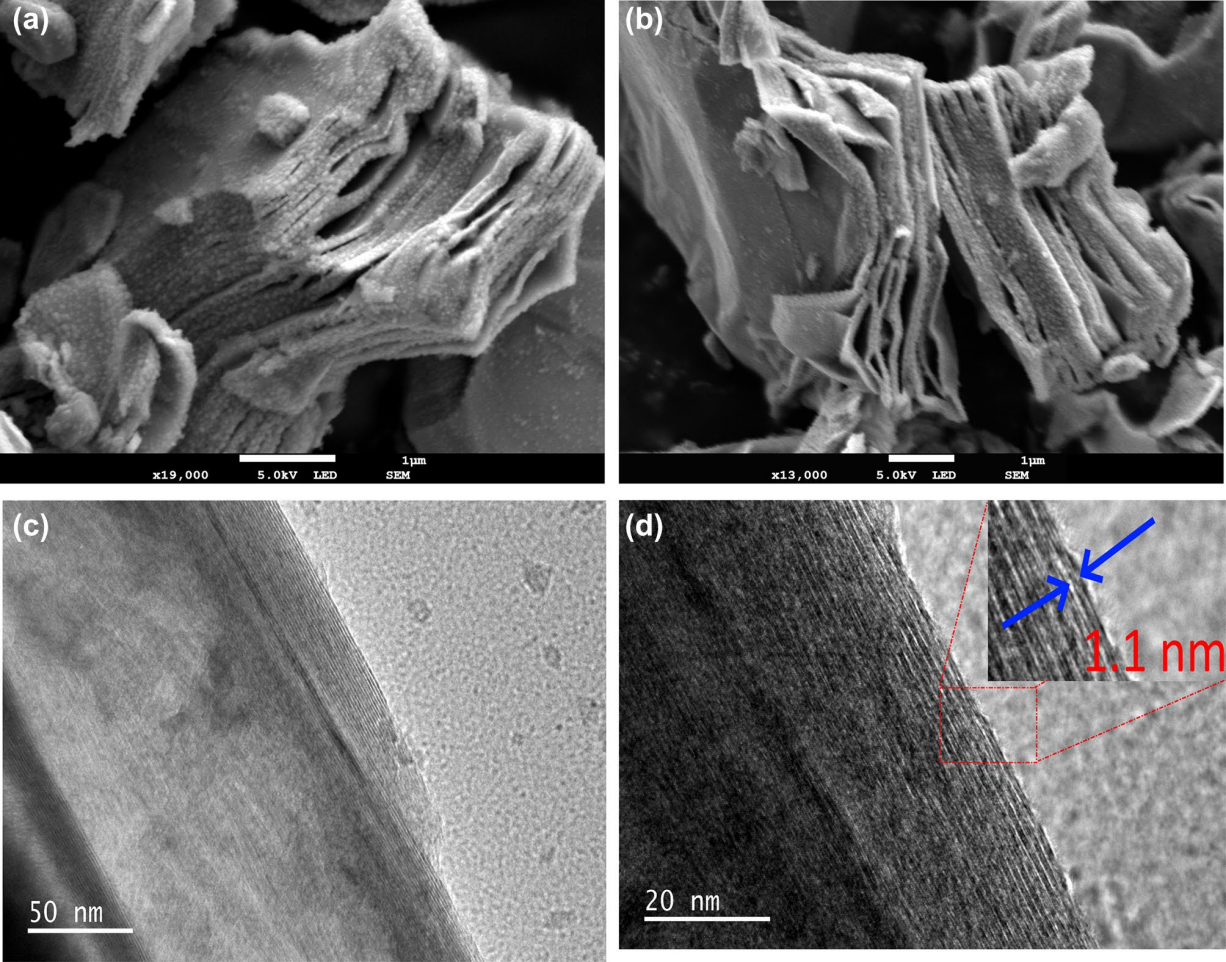
(**a**,**b**) FESEM, and (**c**,**d**) HRTEM images of Mxene (${Ti}_{3}{C}_{2}{T}_{x}$) multilayers. The inset in (**d**) shows the lattice constant of the MXene layer.

Figure 2a, and b demonstrates that the as-synthesized MXene multilayers are multilayered, with particle sizes ranging from 1–10 μm. The layered nature of the MXene is also noticed and shows the thickness of the layered structure is very small and matches previous reports^28,29^. A well-aligned multilayer microstructure, as well as typical accordion-like morphology, and the cross-sectional shear slip of multi-layer MXenes were observed, which are convincing evidence for the exfoliation of ${Ti}_{3}Al{C}_{2}$. Compared to the obviously amorphous and broad (000l) peaks of HF etched MXene, the peaks of ${NH}_{4}{HF}_{2}$-etched ${Ti}_{3}{C}_{2}{T}_{x}$ turned sharp and strengthened, showing a higher crystalline degree and better structural order after exfoliation.

HRTEM photographs of the multilayered Ti_3_C_2_ multilayers are depicted in Fig. 2c, and d and they are in accordance with FESEM images illustrating the multilayered structure of the MXene. Furthermore, the Ti_3_C_2_ multilayers are indicated to be thin, foldable, and flexible which is similar to those of two-dimensional MXenes and graphene^18,30^. The delaminated layers are found to be transparent to the electron beam in TEM. Its Fast Fourier Transform (FFT) represents a hexagonal-based crystal with chain-like features of the nanosheets (Fig. 2d)^30,31^. The estimated lattice constant of the MXene layer is about 1.1 nm as marked in Fig. 2d that shows the presence of Ti_3_C_2_ incubation layers.

**Figure 3:**
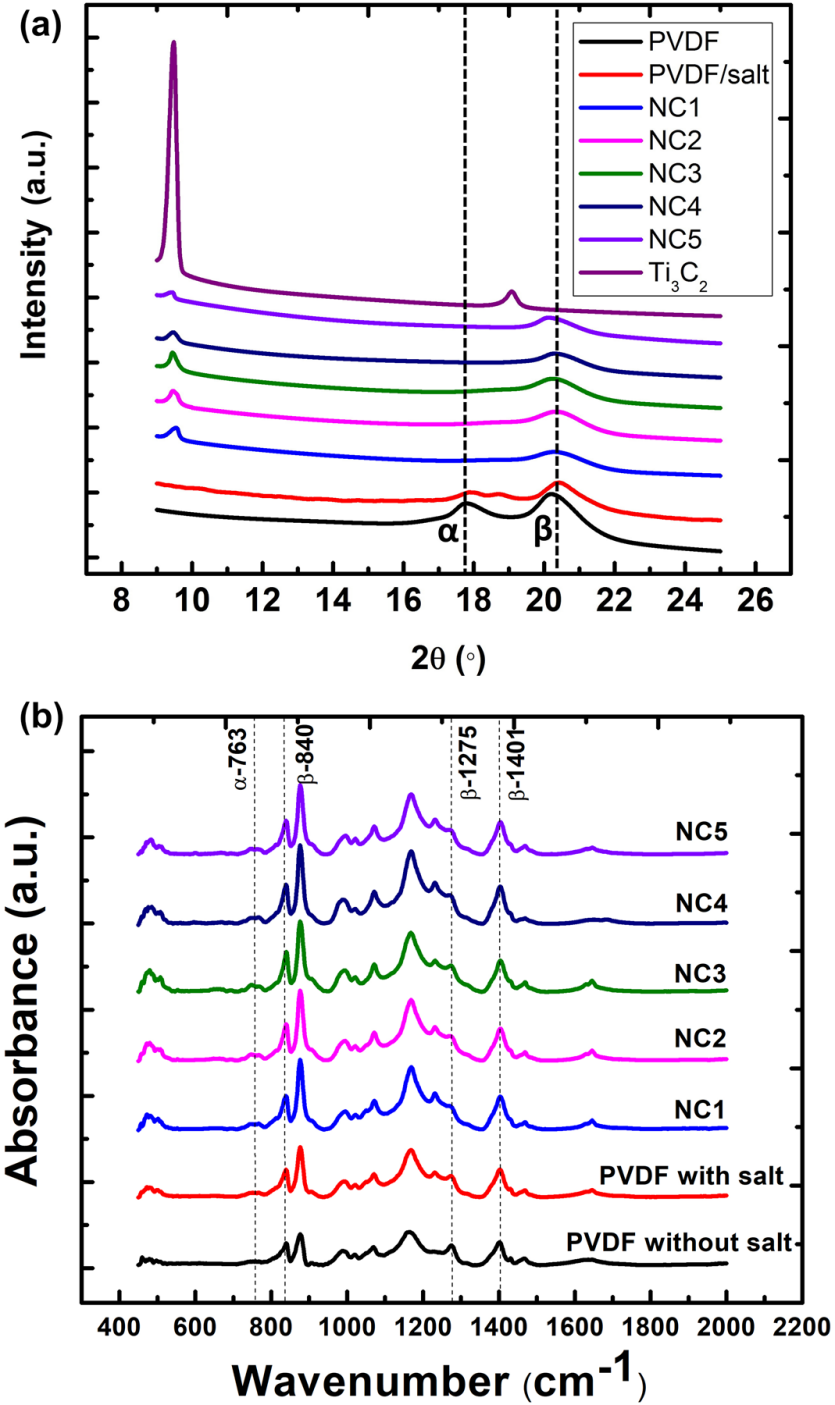
(**a**) XRD results, and (**b**) FTIR measurement of prominent peaks for PVDF, Ti_3_C_2_ nanoflakes, NC1, NC2, NC3, NC4, and NC5 samples.

Figure 3a shows the XRD results of PVDF, Ti_3_C_2_, and nanocomposite for each Ti_3_C_2_ concentration in order to characterize the crystalline phase analysis. As-synthesized Ti_3_C_2_ crystallinity could be detected at 9.5° of NC1, NC2, NC3 NC4 and NC5 that could be likely attributed to the presence of Ti_3_C_2_ as this peak also appeared in the XRD scan of Ti_3_C_2_. PVDF shows prominent peaks at 17.8° and 20.3° which indicate the α- and β-phase content, respectively. Interestingly, the addition of salt in the PVDF matrix has reduced the undesired α-phase at 17.8° as indicated in PVDF/salt sample in Fig. 3a as a result of the arrangement of PVDF chains into trans-state β-phase. Moreover, for all nanocomposites samples, α-phase has been further diminished with the addition of Ti_3_C_2_ multilayers. Hence, PVDF/salt-incorporated Ti_3_C_2_ nanofillers demonstrated the further transformation of the α-phase toward β-phase observed at the peak of 20.3°^28,32,33^.

Figure 3b shows the FTIR spectra of the PVDF and nanocomposite for each Ti_3_C_2_ concentration. The dominance of β-phase is evident from the distinctive bands appearing at 840, 1275, 1401 cm^−1^. The percentage fraction of the $\beta$-phase, $F\left(\beta \right)$ in the PVDF was estimated using the Eq. (1) based on FTIR measurements.

1 $$F\left(\beta \right)=\frac{{A}_{\beta }}{ \left(\frac{{K}_{\beta }}{{K}_{\alpha }}\right){{A}_{\alpha }+ A}_{\beta }} \times 100\%$$

where ${A}_{\alpha }$ and ${A}_{\beta }$ represent measured absorbance at 763 cm^−1^ and 840 cm^−1^, respectively. The values of absorbance coefficients at 763 cm^−1^ and 840 cm^−1^ are K_α_ = 6.1 × 10^4^ cm^2^ mol^−1^ and K_β_ = 7.7 × 10 cm^2^ mol^−1^. The β-fraction of PVDF without salt is 75%, while the addition of salt with PVDF increases it to 77%. On the other hand, β-fractions for NC1, NC2, NC3, NC4, and NC5 are 81, 82, 87, 84, and 83%, respectively. The addition of Ti_3_C_2_ further enhanced the β-fraction of PVDF with the maximum at concentration of 0.03 g/L, and the β-fraction dropped slightly after that.

From the XRD data as plotted in Fig. 3a, the full width half maximum (FWHM) for NC1, NC2, NC3, NC4, and NC5 samples are calculated to be 0.89, 0.88, 0.84, 0.79, 0.76, respectively. Based on the Scherrer formula in Eq. (2), the declining value of FWHM ($$B)$$ indicates the increasing crystallite size ($$\tau$$) of PVDF due to the gradual increase of Ti_3_C_2_ multilayers concentration which yielded enhanced composite crystallinity.

2 $$\tau =\frac{K\lambda }{B.cos\theta }$$

where K = 0.9, and λ = 0.154 nm refer to crystallite shape factor and X-ray wavelength, respectively. The computed values of crystallite size are 9.06 , 9.17, 9.6, 10.2, 10.6 nm for NC1, NC2, NC3, NC4, and NC5, respectively. It could be indicating that the increasing number of crystallite sizes is influenced by the addition of Ti_3_C_2_ concentration with a fixed amount of salt added in our study. The transformation from α- to β-phase was due to the interaction of HMPA and PVDF through formation of intermolecular hydrogen bonds and –OH bonds across the carbon chain to align the –CH_2_ dipoles of PVDF^34^. Meanwhile, the Mg^2+^ cations from Mg-salt addition created the ion–dipole interactions with negative –CF_2_ dipoles of PVDF that stretch the alignment to form stronger β-phase^35^. The addition of Ti_3_C_2_ further enhanced the β-phase due to its –OH termination that reacted with fluorine dipoles of PVDF^23^.

**Figure 4:**
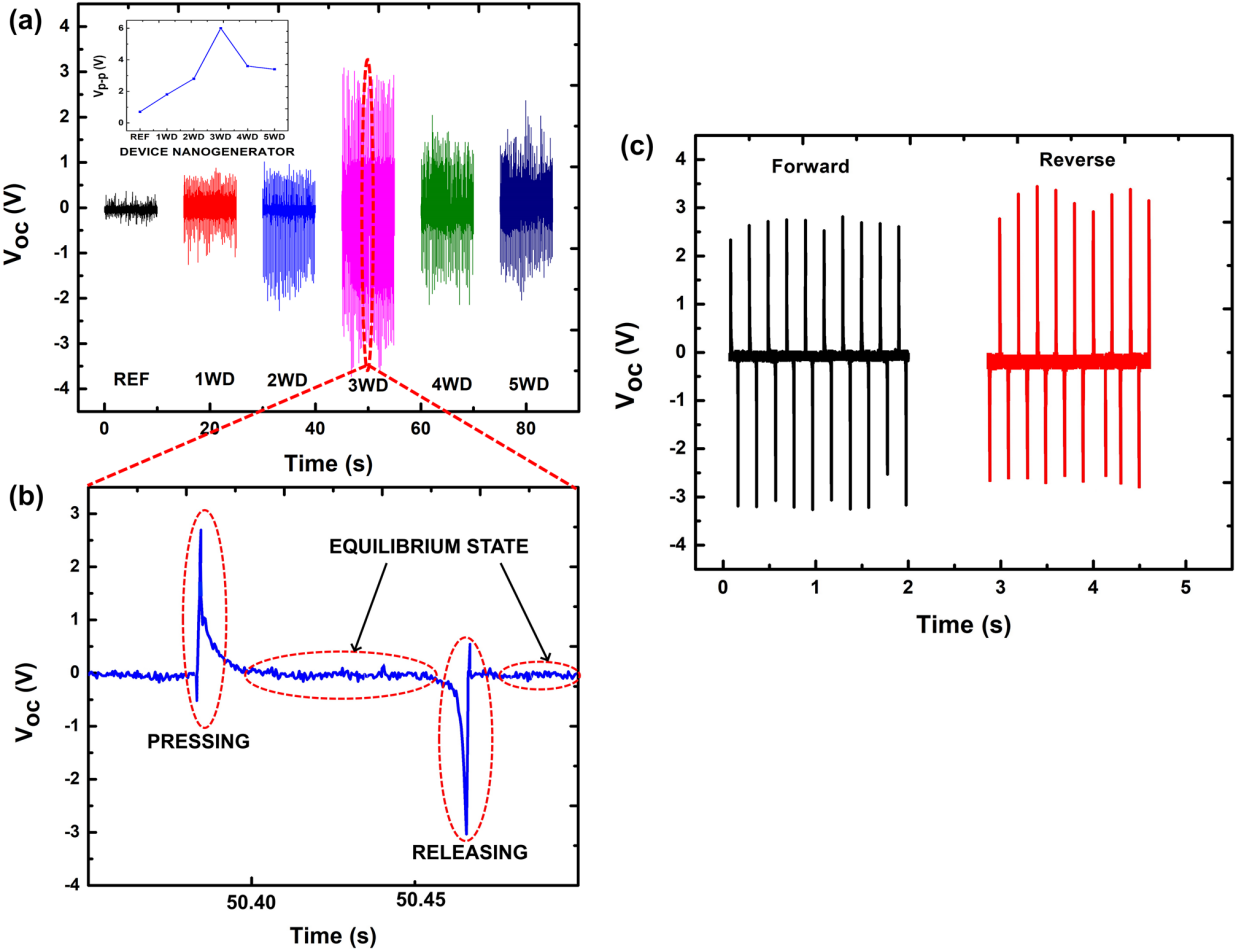
(**a**) *V*_*oc*_ of nanogenerator devices generated by the reference sample, 1WD, 2WD, 3WD, 4WD, and 5 WD. The inset shows the peak-to-peak voltage trend of all nanogenerator devices. (**b**) Magnified view of piezoelectric output voltage behavior for 3WD. (**c**) *V*_*oc*_ of forward and reverse connections of 3WD device.

Figure 4a shows the *V*_*oc*_ measurements for reference devices with salt addition, 1WD, 2WD, 3WD, 4 WD, and 5 WD, respectively. Note that the performance of PVDF without salt has been reported in our previous study^5^. The electrical performance of the fabricated nanogenerator devices was examined by applying 4.7 N at 5 Hz of external stimuli to the devices. The inset in Fig 4 recorded the *V*_*oc*_, which refers to the average peak-to-peak voltage (*V*_*p-p*_) generated by each device. The reference device has the lowest performance of 0.7 V, meanwhile all the nanogenerator devices incorporated with the Ti_3_C_2_ show electrical improvement. The *V*_*oc*_ attained by 1WD, 2WD, 3WD, 4WD, and 5WD are 1.8, 2.8, 6.0, 3.6, and 3.4 V, respectively. It can be observed that the 3WD sample demonstrates the optimum electrical performance and hence it will be the focus of this work to carry out further study. Figure 4b shows the magnified trend of the one-cycle electrical behavior for the 3WD sample. Further elaboration of electrical behavior related to mechanical deformation of pressing and releasing will be explained accordingly. Figure 4c shows the switching polarity test of the 3WD sample. As a consequence of direct piezoelectric effect, 3WD sample shows identical forward and reverse amplitude of *V*_*oc*_ and unlikely to be deduced to contact electrification between the measurement setup and device.

**Figure 5:**
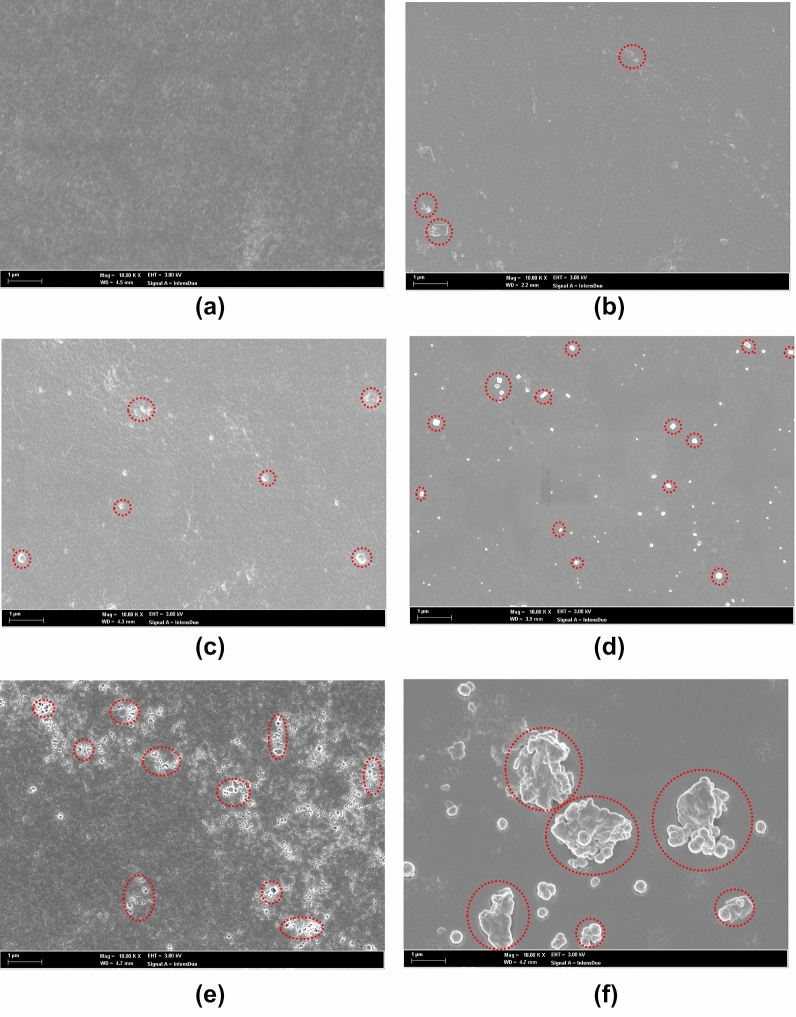
FESEM images of surface morphology (**a**) PVDF, (**b**) NC1, (**c**) NC2, (**d**) NC3, (**e**) NC4, and (**f**) NC5.

To understand the role of Ti_3_C_2_ in the nanogenerator devices, FESEM surface morphology of PVDF, NC1, NC2, NC3, NC4, and NC5 samples were analyzed as shown in Fig. 5a–f, respectively. Figure 5a shows the homogeneous surface of the PVDF layer. Figure 5b–d indicate the appearance of Ti_3_C_2_ multilayers on the surface of the samples as marked in the red circles. The higher amount of Ti_3_C_2_ multilayers could be observed in Fig. 5d of NC3 and it is distributed uniformly as compared to NC1 and NC2. Notably, better electrical performance of 3WD compared to 1WD and 2WD could be owing to a higher concentration of Ti_3_C_2_ in NC3 compared to NC1 and NC2 as can be seen in Fig. 5d, b, and c, respectively. However, as the concentration of Ti_3_C_2_ increases in NC4 and NC5 as shown in Fig. 5e,f, agglomeration of Ti_3_C_2_ is found. To confirm the presence of Ti_3_C_2_ in NC1 to NC5 samples, the energy dispersive X-ray (EDX) analysis and elemental mapping of the Ti_3_C_2_ had been conducted and provided in Figure S1 and S2 of Supplementary Information. It is found that Ti and C elements are dominant when in high concentration samples because of the high viscosity of Ti_3_C_2_ in the PVDF solution. Therefore, the agglomeration reduces the distribution of Ti_3_C_2_ in the PVDF matrix and disfavor enhancing the β-phase. The poor performance in 4WD and 5WD samples might be caused by the agglomeration of Ti_3_C_2_ multilayers in PVDF that impede the nucleation of β-phase and hence the polarization of PVDF molecular chains^3^. Agglomeration of Ti_3_C_2_ is reported to result in low uniformity distribution of fillers in PVDF matrices and hence create randomness among the aligned dipole domain areas and lead to minimum dipole alignment of Ti_3_C_2_ in the PVDF matrix^17,36,37^. Even though NC4 and NC5 show better crystallinity, nevertheless NC3 has the dominant optimum β-phase that defeats the effect of enhanced composite crystallinity^38^. The agglomeration of Ti_3_C_2_ tends to hinder the rotation of the PVDF molecular chain into all-trans planar zigzag chain configuration of β-phase content. This can be further verified by XRD and FTIR measurements as shown in Fig. 3, that NC3 demonstrates the highest β-peak compared to other samples indicating the optimum improvement of PVDF crystallinity besides the salt addition in PVDF. The improved crystallinity of the NC3 sample is likely associated with homogeneous dispersion of 0.03 g/L Ti_3_C_2_ and consequently higher interfacial interaction between nanofiller and PVDF matrix.

**Figure 6:**
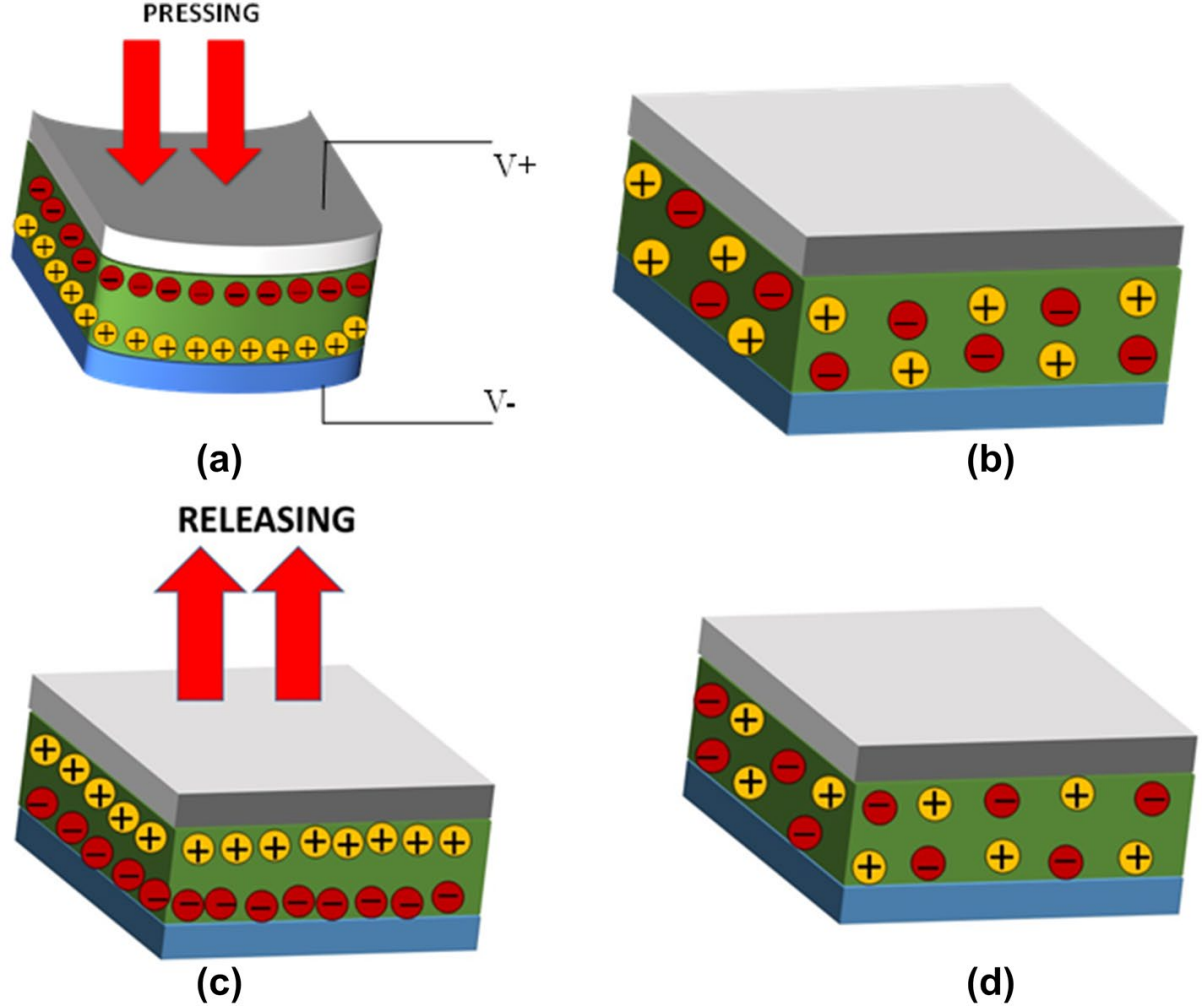
Transformation of mechanical energy into electrical energy ascribed to (**a**) pressing, (**b**) equilibrium state, (**c**) releasing, and (**d**) initial equilibrium state.

**Figure 7:**
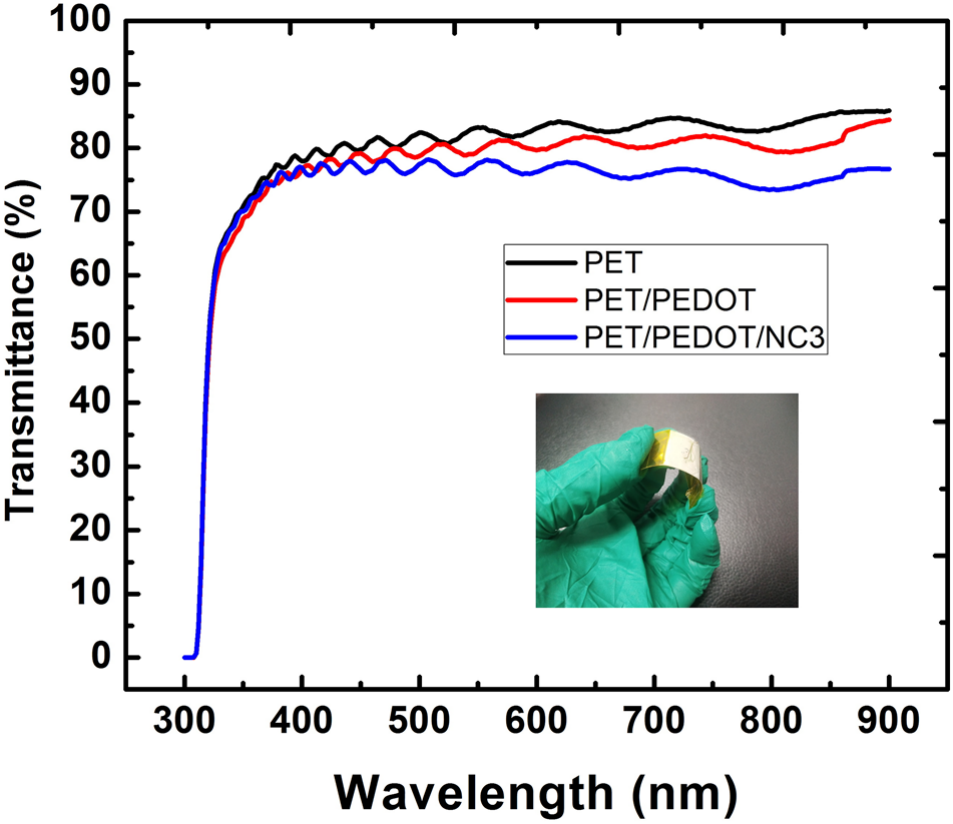
Optical transparency measurements of the devices in the visible region.

Figure 6 depicts the possible mechanical energy harvesting mechanism of piezoelectric nanogenerator devices deduced to tapping action into electrical energy. In Fig. 6a, when the device experiences the pressing stress, it is receiving mechanical stimuli. Subsequently, negative and positive charges are separated in opposite directions toward the top and bottom electrodes, respectively. The acquired net charge from the difference in top and bottom electrodes is ascribed to the polarization effect that will create an electric field. Hence, the free charges from the AgNWs electrode flow to the opposite PEDOT:PSS electrode through the external circuit and form a positive signal as shown in Fig. 4b. Meanwhile, the high amount of free charges flowing from AgNW to PEDOT:PSS could be a result of high difference of electronegativity from both electrodes which originate from Ti_3_C_2_ property^39^. As there is no additional stress being applied to the device, all the charges are in an equilibrium state and resulting in zero potential difference across the electrodes as shown in Fig. 6b. In Fig. 6c, when the external pressure is released from the device, the free charges in the PEDOT:PSS electrode flow in the reverse direction toward the AgNW electrode and form a negative signal as shown in Fig. 4b. When there is no external stress as shown in Fig. 6d, the device returns to its initial equilibrium state again. Figure 4b recorded zero potential of the 3WD sample after the negative signal. Figure 7 shows the transparency test of PET, PVDF/PEDOT:PSS/PET, and 3WD sample without the top electrode in the visible region from 380 to 700 nm of wavelength. The transparency measurements were conducted relative to the air. The blank PET sample shows the highest transparency range from 80 to 86%. As predicted, PET coated with PEDOT:PSS and 3WD show a slight reduction of transparency varying from 78 to 85% and 74% to 76%, respectively. Note that the lowest transparency of the 3WD sample may be related to the addition of Ti_3_C_2_ flakes. The inset in Fig. 7 shows the flexible picture of the fabricated 3WD sample.

**Figure 8:**
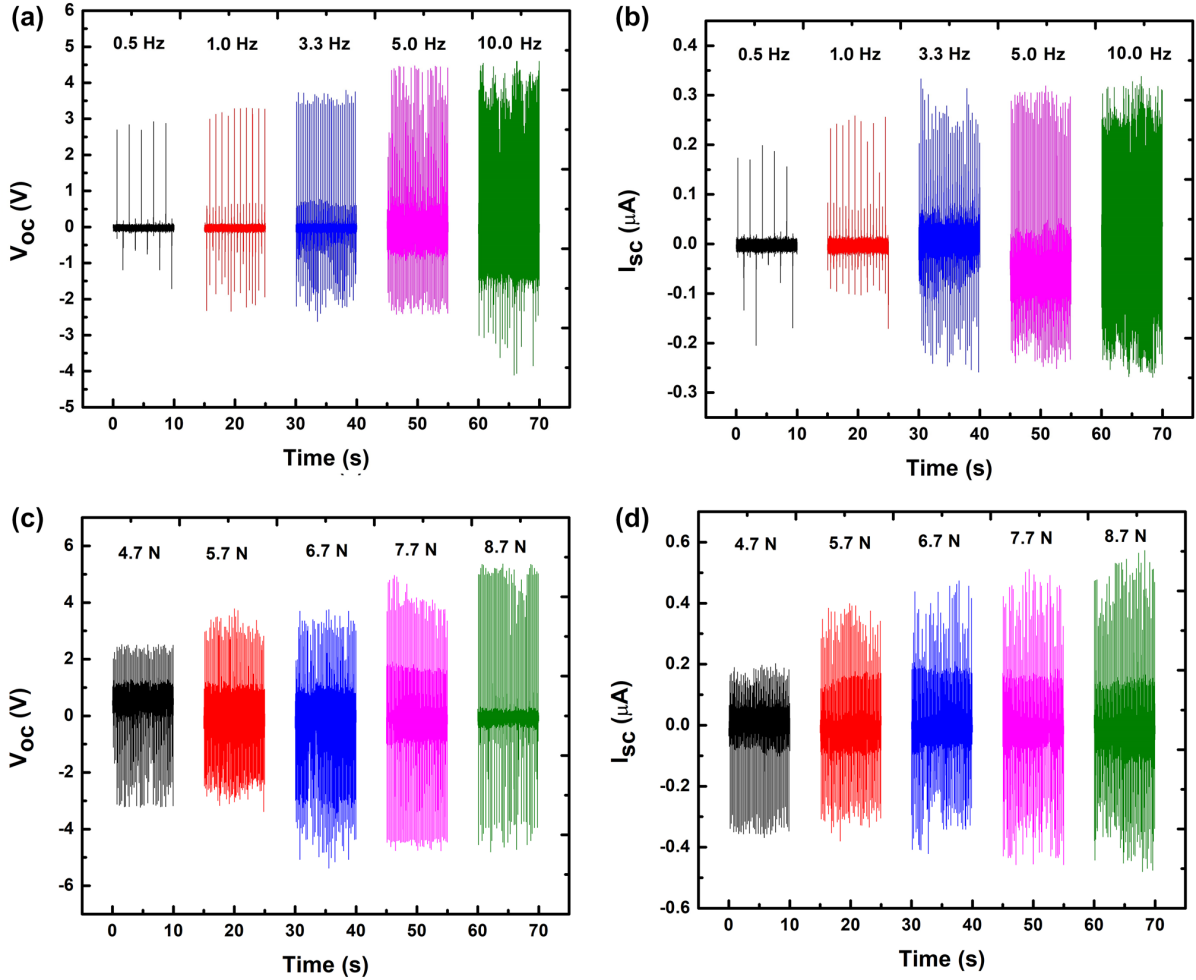
Generated *V*_*oc*_ and *I*_*sc*_, at (**a**,**b**) different tapping frequencies and (**c**,**d**) tapping forces, respectively by 3WD devices.

Figure 8 shows the further electrical measurements of the 3WD sample in order to figure out its performance under different tapping frequency and force. Figure 8a, and b show the plots of *V*_*oc*_ and *I*_*sc*_ versus time, respectively at low frequency as exhibited by human or animal motion. When the device is tapped under the frequency ranging from 0.5 to 10 Hz at a constant tapping force of 4.74 N, it can be seen that *V*_*oc*_ is gradually increasing as the frequency gets higher. The obtained *V*_*oc*_ was in the trend of 3.8, 5.1, 5.5, 6.0, and 6.9 V for 0.5, 1.0, 3.3, 5.0, and 10.0 Hz, respectively. Similarly, *I*_*sc*_ shows the increasing trend as well, estimated to be 0.30, 0.35, 0.51, 0.52, and 0.53 µA under the same applied frequency. The higher electrical signals with the increasing tapping frequency might be ascribed to the vibrational motion of the material approaching the resonance frequency of piezoelectric material. At the resonance frequency, piezoelectric generates maximum electrical output^40^. Likewise, in Fig. 8c, and d, it is observed that the electrical performance of the fabricated device also increases with higher applied force. *V*_*oc*_ is increased significantly from 5.7, 6.5, 8, 8.2 to 8.9 V when the tapping pressure is applied higher gradually from 4.7, 5.7, 6.7, 7.7 to 8.7 N, respectively at 5 Hz. The exerted force was deliberately set in the range from 4.7 to 8.7 N to mimic the force that can be generated daily by animals (hamster, dog, and cat) to human mechanical motion (walking, typing, and running)^41^. Meanwhile, the equivalent *I*_*sc*_ is recorded to be 0.52, 0.69, 0.72, 0.8, 0.89 µA. The obtained electrical data indicate that harvested electrical energy is directly proportional to the applied force on the nanogenerator^42^.

**Figure 9:**
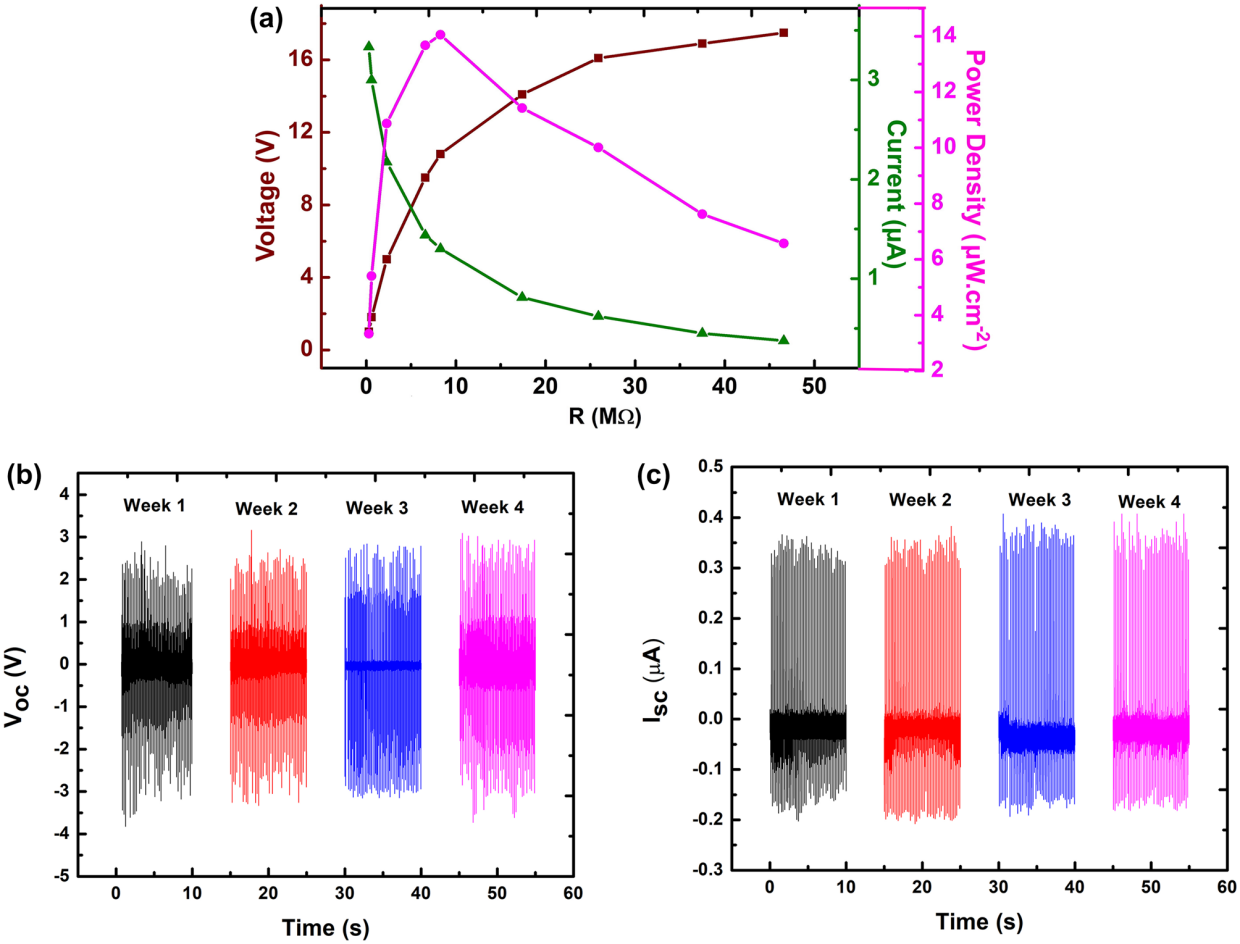
(**a**) Power density estimation based on the obtained *V*_*oc*_ and *I*_*sc*_ over different resistance values, and (**b**,**c**) the electrical stability test for 3WD devices.

Figure 9a shows the voltage, current, and instantaneous output power density of nanogenerator 3WD as a function of various load resistors ranging from 0.3 to 46.6 MΩ. The measured voltage shows an increasing pattern with the increment of the load resistor while the current displays the opposite trend. The maximum power density that can be achieved by the all-solution process fabrication technique in this work is about 14 µW.cm^−2^ at 10.8 MΩ. The generated power density is considerably compatible with the different fabrication methods of 2D material-based nanogenerators as shown in Table 1. Remarkably, even though our piezoelectric layer is relatively thinner, the power density could attain one-fourth of thicker PVDF fiber sample prepared by Yadav et al.^36^. Besides, the stability test was also carried out 4 weeks consecutively as shown in Fig. 9b, and c. The performance of the 3WD device shows high durability and endurance as there is no distinctive deterioration observed throughout the weekly measurements. Practical application of the fabricated 3WD device as a power source for low-power electronic devices was provided in Supplementary Video 1. The electrical energy of the nanogenerator is rectified by a full-wave bridge rectifier circuit and then stored to the 2.2 µF of a capacitor with the setup reported in our previous work^43^. The 3WD device charged up the capacitor for 45 s in order to attain 3.1 V of the voltage source prior to being able to light-up a LED. Therefore, nanogenerators incorporated with Ti_3_C_2_ multilayers demonstrated their potential in energy harvester applications throughout daily mechanical activities.

**Table 1:** Reported power density achievement of various nanomaterial-based piezoelectric nanogenerators.

No	References	Composites	Area of device (cm^2^)	*V*_*oc*_*/I*_*sc*_	Fabrication Method	Power Density (µW. cm^−2^)
1	Yadav et al.^36^	hBN/PVDF fiber	1 × 3	68.0 V/0.1 µA	Electrospun	53.2
2	Shetty et al.^3^	Talc/PVDF nanofiber	1 × 6	9.1 V/0.0165 µA	Electrospun	1.12
3	Wu et al.^8^	MoS_2_/PMMA	2 × 2.5	0.015 V/0.00002 µA	Seed-free CVD	0.2
4	Yaqoob et al.^17^	BaTiO_3_/n-Gr/PVDF	2 × 4	10.0 V/2.5 µA	Solution-processed	5.8
5	Karan et al.^44^	Fe-doped RGO/PVDF	2 × 3	5.1 V/0.25 µA	Solution casting	–
6	Kar et al.^45^	SnO_2_/PVDF	1.6 × 2.5	42 V	Solution casting	~ 2.45
7	Wang et al.^23^	MXene/PVDF TrFE	1.2 × 1.2	1.5 V/0.15 µA	Electrospinning	0.364
8	This Work	Ti_3_C_2_/PVDF	1.5 × 2	6.0 V/0.52 µA	All-solution processed	14

## Conclusion

The 2D Ti_3_C_2_ multilayers were synthesized using a wet-etching chemistry process and it was incorporated in PVDF matrix to fabricate the piezoelectric nanogenerator by all-solution mean deposition. Spin- and spray-coating methods were conducted to deposit bottom and top electrodes as well as the nanocomposite mixture consisting of PVDF, MgCl_2_, and Ti_3_C_2_ multilayers. XRD measurement confirms the formation of β-phase and diminishing α-phase at different concentrations of Ti_3_C_2_ in the PVDF. A 3WD nanogenerator device with a 0.03 g/L concentration of Ti_3_C_2_ has attained the highest *V*_*oc*_ of about 6.0 V as compared to other fabricated devices in this work. The optimum performance of the nanogenerator may be probably caused by the homogeneous dispersion of Ti_3_C_2_ multilayers in the PVDF matrix that is observed in its surface morphology characterization. Uniform dispersion of Ti_3_C_2_ would enhance the formation of β-phase and promote the dipole moment alignment of piezoelectric material. The fabricated 3WD device has also demonstrated the ability to transform mechanical energy to electrical energy at low frequency and force, ranging from 0.5 to 10 Hz, and 4.7 to 8.7 N, respectively. A calculated power density of the 3WD nanogenerator is about 14 µW.cm^−2^ at 10.8 MΩ which is notably high among the reported nanogenerator performance. Additionally, the electrical performance of the optimized device is stable for up to 4 weeks consecutively without significant degradation and able to light up the LED. Therefore, all-solution-processed energy harvesters with the synergistic effects by blending Ti_3_C_2_ multilayers, PVDF, and salt using high polar HMPA solvent demonstrated the potential as an energy source for low power electronic devices applications.

## Supplementary Information

Supplementary Information 1.

Supplementary Video 1.
