# Supplementary material for: Exploration of 2D Ti3C2 MXene for all solution processed piezoelectric nanogenerator applications
Source: Sci Rep. 2021 Aug 31;11:17432. doi: 10.1038/s41598-021-96909-0 (PMC8408174; doi:10.1038/s41598-021-96909-0)
Supplement: Supplementary file 1 — Supplementary Information 1. [file 41598_2021_96909_MOESM1_ESM.docx]

Supplementary Information

Exploration of 2D Ti_3_C_2_ MXene for All Solution Processed Piezoelectric Nanogenerator Applications

Rahmat Zaki Auliya^1^, Poh Choon Ooi^1,*^, Rad Sadri^2^_’_^*^, Noor Azrina Talik^3^, Zhi Yong Yau^1, 4^, Muhammad Aniq Shazni Mohammad Haniff^1^, Boon Tong Goh^3^, Chang Fu Dee^1,*^, Navid Aslfattahi^5^, Sameer Al-Bati^6^, Khatatbeh Ibtehaj^6^, Mohammad Hafizuddin Hj Jumali^6^, M.F. Mohd Razip Wee^1^, Mohd Ambri Mohamed^1^, Masuri Othman^1^

^1^Institute of Microengineering and Nanoelectronics, Universiti Kebangsaan Malaysia, 43600, Bangi, Selangor, Malaysia

^2^ Faculty of New Sciences and Technologies, University of Tehran, Tehran, Iran

^3^Low Dimensional Materials Research Centre (LDMRC), Department of Physics, Faculty of Science, University of Malaya, 50603, Kuala Lumpur, Malaysia

^4^Lee Kong Chian Faculty of Engineering & Science, Universiti Tunku Abdul Rahman, Sungai Long Campus, Jalan Sungai Long, Bandar Sungai Long, Cheras, 43000 Kajang, Selangor, Malaysia

^5^Department of Mechanical Engineering,

Faculty of Engineering, University of Malaya, 50603, Kuala Lumpur, Malaysia

^6^School of Applied Physics, Faculty of Science and Technology, Universiti Kebangsaan Malaysia, 43600 UKM Bangi, Selangor, Malaysia

*Correspondence to [[pcooi@gmx.com](mailto:pcooi@gmx.com)] or [[cfdee@ukm.edu.my](mailto:cfdee@ukm.edu.my)] or [[rad.sadri@gmail.com](mailto:rad.sadri@gmail.com)]


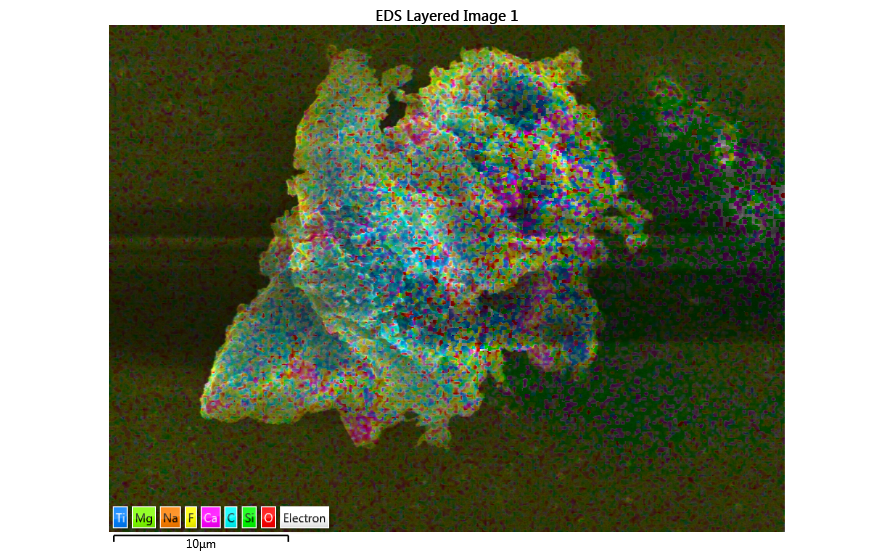


Figure S1. Elemental mapping of Ti_3_C_2_ nanoflakes.


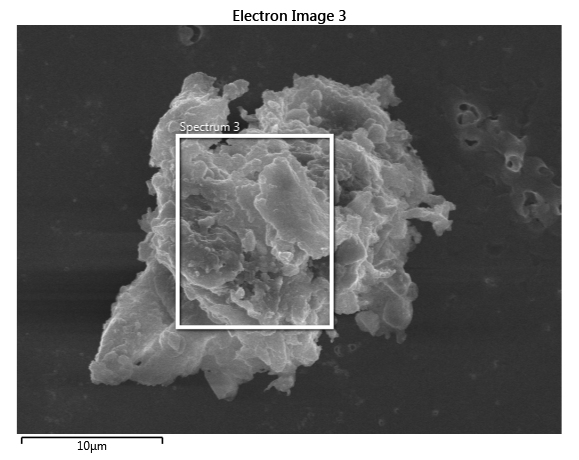


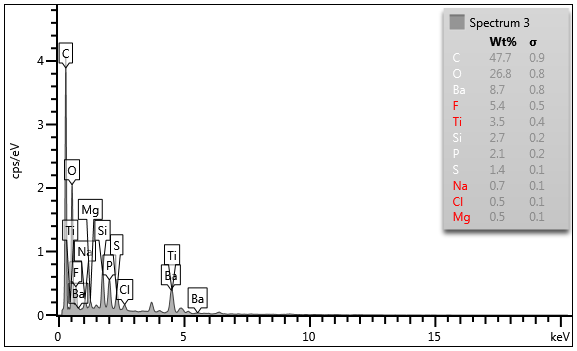
(a)

(b)

Figure S2. (a) FESEM EDX image, and (b) intensity for each element of Ti_3_C_2_ nanoflakes.
